# Development of a 3D printed phantom for commissioning and quality assurance of multiple brain targets stereotactic radiosurgery

**DOI:** 10.1007/s13246-023-01374-w

**Published:** 2024-01-29

**Authors:** Godfrey Mukwada, Andrew Hirst, Pejman Rowshanfarzad, Martin A. Ebert

**Affiliations:** 1 Department of Radiation Oncology, Sir Charles Gairdner Hospital, Hospital Ave, Nedlands, WA Australia; 2 School of Physics, Mathematics and Computing, University of Western Australia, Crawley, WA Australia; 3 Medical School, Australian Centre for Quantitative Imaging, University of Western Australia, Crawley, WA Australia; 4 School of Medicine and Population Health, University of Wisconsin, Madison, WI USA

**Keywords:** 3D printing, PLA stoneFil, PLA marble, Multiple brain targets

## Abstract

Single plan techniques for multiple brain targets (MBT) stereotactic radiosurgery (SRS) are now routine. Patient specific quality assurance (QA) for MBT poses challenges due to the limited capabilities of existing QA tools which necessitates several plan redeliveries. This study sought to develop an SRS QA phantom that enables flexible MBT patient specific QA in a single delivery, along with complex SRS commissioning. PLA marble and PLA StoneFil materials were selected based on the literature and previous research conducted in our department. The HU numbers were investigated to determine the appropriate percentage infill for skull and soft-tissue equivalence. A Prusa MK3S printer in conjunction with the above-mentioned filaments were used to print the SRS QA phantom. Quality control (QC) was performed on the printed skull, film inserts and plugs for point dose measurements. EBT3 film and point dose measurements were performed using a CC04 ionisation chamber. QC demonstrated that the SRS QA phantom transverse, coronal and sagittal film planes were orthogonal within 0.5°. HU numbers for the skull, film inserts and plugs were 858 ± 20 and 35 ± 12 respectively. Point and EBT3 film dose measurements were within 2.5% and 3%/2 mm 95% gamma pass rate, respectively except one Gross Tumour Volume (GTV) that had a slightly lower gamma pass rate. Dose distributions to five GTVs were measured with EBT3 film in a single plan delivery on CyberKnife. In conclusion, an SRS QA phantom was designed, and 3D printed and its use for performing complex MBT patient specific QA in a single delivery was demonstrated.

## Introduction

The single plan technique (SPT) for brain multiple targets (MBT) stereotactic radiosurgery (SRS) is becoming widely used due to its time-saving benefits in planning and delivery [1–5]. This technique involves a high dose prescription and tight target margins which requires comprehensive and stringent quality assurance (QA) measures. The availability of QA phantoms capable of accommodating MBT cases is limited as the treatment plan must be delivered several times to accommodate all targets. Re-delivery of the same plan takes time and resources that can be used to improve clinical workflow and patient care. The emergence of 3D printing technology provides the opportunity to fabricate an SRS QA phantom that enables the assessment of multiple targets using a single plan delivery.

3D printing, also known as Additive Manufacturing (AM), was invented in the 1980s and gained significant attention from the Physical Science community in the past decade [6]. The widespread utilization of 3D printing technology has been fuelled by the availability of various 3D printers, their affordability, and the growing demand for its applications [6–10]. Different filament types with distinct characteristics are available, and these characteristics can be further expanded by adjusting printer settings. Different densities in the printed objects can be achieved by using filaments of different densities and changing fused deposition modelling such as infilling percentage, extrusion rate and through temperature control [8, 9, 11–15]. Ideally, phantoms should have radiation tissue equivalence as recommended by ICRU report 44 [16].

The aim of this study was to design and fabricate a robust SRS QA phantom that enables film, ionisation chamber and microdiamond dose measurements in a patient geometry. Prior research conducted by Pereira et al. demonstrated that 100% infill polylactic acid (PLA) standard and coloured meets the ICRU report 44 recommendations of equivalent tissue materials [17]. The report [16] recommends evaluation of materials chemical composition, mass attenuation coefficient, stopping power, and mass density. Investigation by Kairn et al. also demonstrated that PLA StoneFil with 100% infill can serve as a suitable material with bone-equivalent material characteristics [18]. In line with these previous studies, the present research aims to build upon their findings rather than examining or assessing tissue or bone equivalent materials according to the guidelines outlined in ICRU report 44. The focus is on developing a phantom specifically tailored for SRS QA in the context of MBT.

## Materials and methods

### Filament assessment and 3D printer

The SRS QA phantom was produced using a Prusa i3 MK3S 3D printer (Prusa Research, Prague, Czech). This printer, equipped with a single nozzle, was utilized in conjunction with two types of PLA filament for the printing process. Based on previous work and experience in our department, PLA marble was chosen as a suitable substitute for brain/soft tissue. Extensive studies performed on PLA standard and various coloured PLA filaments [17] have demonstrated their comparable radiation interaction characteristics. Consequently, PLA marble was bench marked against PLA black. To assess the characteristics of the printed PLA marble and black materials, two sets of six 2 × 2 × 2 cm^3^ cubes were fabricated using the 3D printer. Subsequently, the cubes were scanned using a Toshiba Aquilion CT scanner (Canon Inc., Tokyo, Japan) following SCGH SRS scanning protocol (1 mm slice thickness, large field of view, FC9 reconstruction algorithm). The 3D Printer settings are shown in Table 1. The HU values of the scanned cubes were quantitatively evaluated using ImageJ software (National Institutes of Health, Bethesda, Maryland, U.S.).

After confirming that PLA marble was equivalent to PLA black in terms of HU numbers, the percentage infill that resulted in equivalent brain tissue was evaluated using the HU assessment method. Small cubes of size, 2 × 2 × 2 cm^3^ were printed with different filament percentage infill i.e., 90, 91, 92, 93, 94, 95 and 96. A plot was generated to compare the filament percentage infill with the average HU numbers of brain tissue. The same process was repeated with PLA stoneFil concrete (FormFutura, BV, Nijmegen, Netherlands) to establish skull equivalence [18].

The HU numbers for the skull and brain were obtained by averaging 15 regions of interest (ROI) over 3 randomly selected slices from each scan. The CT images were imported into ImageJ software and the HU values for 6 × 6 mm^2^ ROIs were measured. This process was performed on two real adult heads and the CIRS stereotactic end-to-end verification (STEEV) phantom (CIRS Inc. FL, USA). However, due to differences in design, the HU values for STEEV skull were determined in the Eclipse TPS ver. 16.1 (Varian, Palo Alto, USA), using a point of interest method. The same procedure using ROI was also applied to the printed SRS QA phantom.

Although, the aim was not to assess tissue or bone equivalent materials according to the guidelines outlined in ICRU report 44, simple geometry measurements were performed. Slabs of PLA and StoneFil were 3D printed and point dose measurements were performed at 8 cm equivalent depth at 100 cm source axis distance (SAD) for a 5 cm × 5 cm field size using IBA CC04 ionisation chamber (IBA Dosimetry GmbH, Schwarzenbruck, Germany). These measurements were then compared with those obtained at the same depth in RW3 slab phantom (PTW Freiburg GmbH, Germany).

**Table 1:** Printing parameters

Parameters	Skull	Brain inserts/soft tissue	
		Plug inserts for point dose measurement	Film inserts
Material	PLA StoneFil	PLA marble	PLA marble
Nozzle size (mm)	0.4	0.4	0.4
Filament diameter (mm)	1.75	1.75	1.75
Layer thickness (mm)	0.2	0.2	0.2
Nozzle temperature (°C)	215	215	215
Printing bed temperature (°C)	60	60	60
Fill pattern	Rectilinear	Rectilinear	Rectilinear
Infill (%)	100	93	83
Infill angle (0)	45	45	45
Maximum print speed (internal fill) (mm/s)	80	80	80
Print speed (outside perimeter) mm/s	25	25	25
Print speed (perimeter) mm/s	25	25	25
Maximum volumetric speed (mm^3^/s)	8	8	8

### SRS QA phantom design and production

**Fig. 1:**
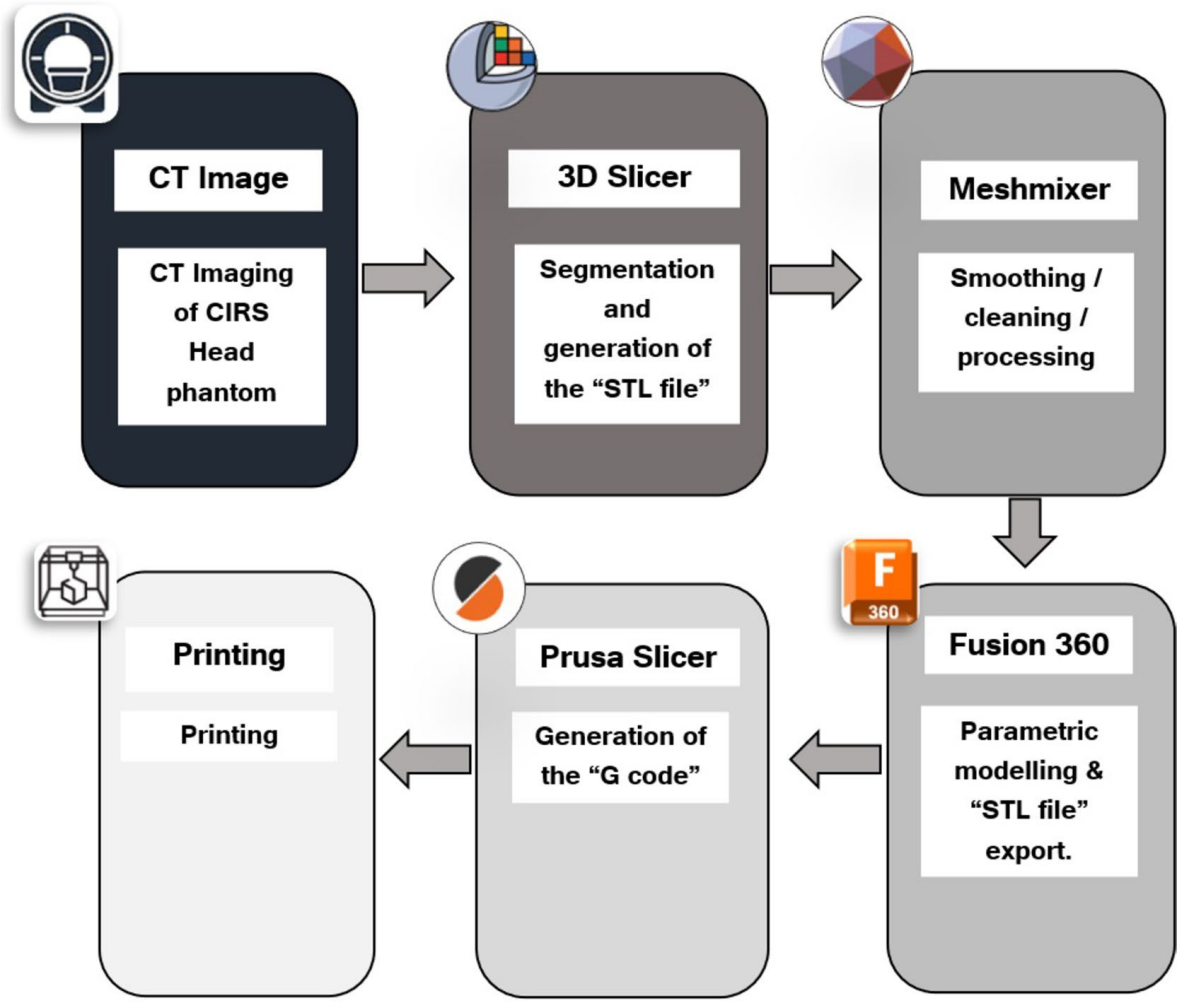
Phantom formation flowchart

A flowchart of the design and production of the SRS QA phantom is shown in Fig. 1. The STEEV phantom was imaged on a Toshiba Aquilion CT scanner following the SCGH SRS scanning protocol. The CT scan was imported into 3D slicer software for segmentation and generation of “STL file”. This file was then imported into Meshmixer (Autodesk Inc, San Rafael, USA), for manual editing tasks such as removing erroneous features and filling holes. The cleaned and smoothed model in “STL file” format was subsequently exported to Fusion 360, where parametric modelling took place. After completing the parametric modelling stage, the STL file was exported to Prusa Slicer software, which generated the “G code” for 3D printing on the Prusa i3 MK3S printer.

**Fig. 2:**
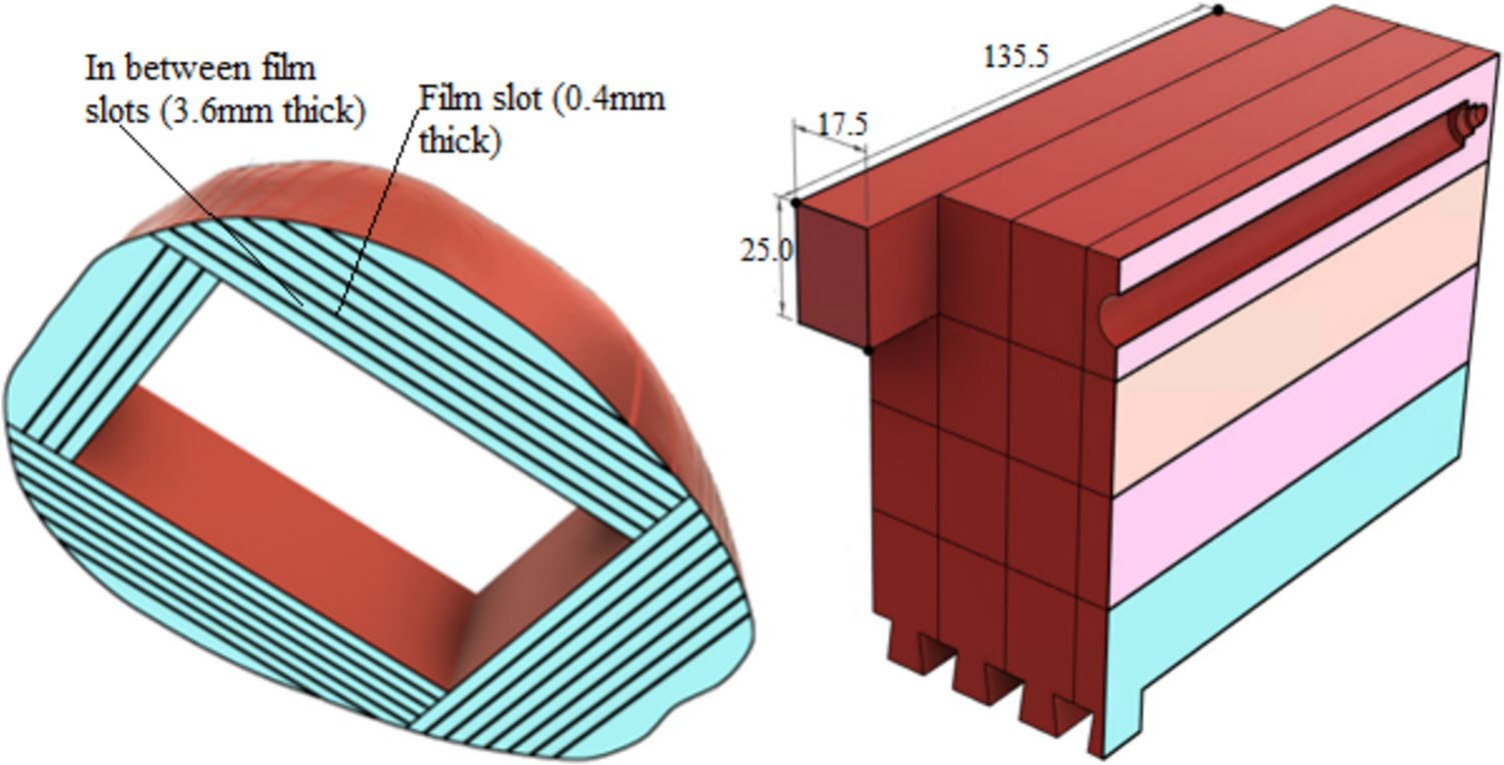
Transverse cross-session of the brain equivalent tissue, showing dimensions of the film slots. Additionally, a sagittal cross-section showing the chamber insert and the dimensions of the plug. All plug dimensions are measured in mm

The point dose measurement plugs, and film insert dimensions are as shown in Fig. 2. The film slot thickness of 0.4 mm was chosen to accommodate the EBT3 film, which has a thickness of 0.278 mm, while also allowing for a small gap to minimize the risk of film scratching. In addition, the phantom was designed with a dedicated base to facilitate immobilisation and ensure easy and reproducible setup. The bottom plugs feature legs (see Fig. 2) that firmly locks into the phantom base. The base itself is equipped with three legs, which enable levelling using a small spirit level for accurate alignment. To allow a practical tight fit, adjacent faces were designed with 0.1 mm tolerances and post processing (sanding) was performed to achieve a smooth finish.

### SRS QA phantom quality control

EBT3 films were inserted into the film inserts and the phantom was scanned using the Toshiba Aquilion scanner following the department’s SRS scanning protocol. The EBT3 films were precisely cut using a Beambox 40 laser cutter (Flux Australia, Campbellfield, Victoria, AU) to ensure a snug fit into the respective film inserts. HU numbers were assessed for the film inserts and reprinted if the average HU deviated by ± 20 HU from 35HU. The skull was also evaluated by scanning and assessing the HU numbers.

The SRS QA phantom, equipped with complete film inserts, was CT scanned and tested for alignment and orthogonality of the film planes (transverse, coronal and sagittal). In the case of CyberKnife, the deliverability of the QA plan relies on the system’s ability to track the phantom. Tracking and deliverability were successfully tested by creating a simple plan on Precision ver. 2.0.1.1 (Accuracy Inc. Sunnyvale, CA, USA) and delivering it on the CyberKnife system.

### End to end testing of the SRS Phantom

The SRS QA phantom was scanned following the SRS CT protocol, and the acquired images were imported into the Eclipse TPS for contouring and treatment planning. A plan with four Gross Tumour Volumes (GTVs) was generated and exported to a Varian Truebeam linear accelerator (Varian, Palo Alto, USA) for delivery. To validate the application of the phantom, a single point reference dose was measured at the centre of the GTV using an IBA CC04 ionisation chamber. The measurement was performed following the dosimetry protocol outlined by IAEA TRS 483 [19].

The contoured SRS QA phantom from Eclipse (as mentioned above) was exported to the Precision TPS as a QA phantom. A 6 MBT plan was then mapped onto the SRS QA phantom using rigid registration. Dose calculations were performed following the department CyberKnife SRS QA protocol. For each GTV, unexposed EBT3 films (Ashland Inc. Wayne, NJ USA) were inserted into the corresponding film slots while the other EBT3 film slots had exposed EBT3 film inserted. The films were processed using an Epson 12000XL scanner and analysed using SNC patient software ver. 8.0.0.1 (Sun Nuclear Corporation, FL, USA). Gamma analysis was performed with pass criteria set at 3% dose difference and 2 mm distance-to-agreement, with a 95% pass rate at 10% threshold. The measured dose distributions from the films were compared with the dose distributions calculated by the TPS.

## Results

### Filament assessment

**Fig. 3:**
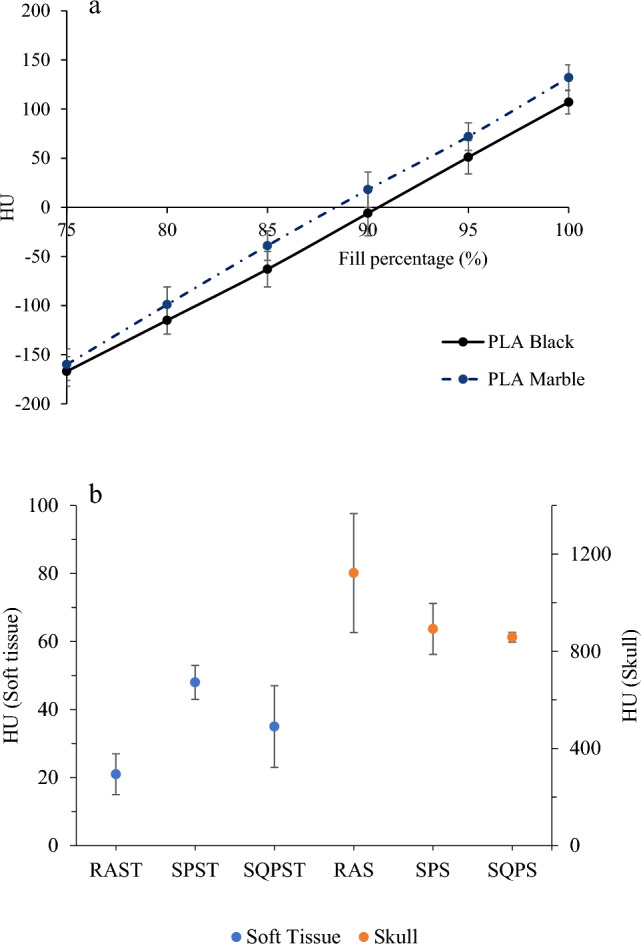
**a** Validation PLA marble against PLA black. **b** Comparison of HUs for real adult soft tissue (RAST), STEEV phantom soft tissue (SPST), SRS QA phantom soft tissue (SQPST), real adult skull (RAS), STEEV phantom skull (SPS) and SRS QA phantom skull (SQPS)

The mean difference between the HU numbers of PLA marble and black was found to be 20 ± 7 (1 SD). PLA marble consistently exhibited higher HU values compared to PLA black, as shown in Fig. 3a. Based on the investigations, the skull was printed with PLA stoneFil at 100% infill, while the point dose measurement plugs and film inserts were printed with 93% and 83% infill, respectively. The determination of the 83% infill for the film inserts was obtained through trial and error. The average HU for the SRS QA phantom soft tissue was 35 ± 12 (1 SD) HU. The soft tissues of both the real adult head and the STEEV phantom exhibited comparable HU values. The average HU for the SRS QA phantom skull was 858 ± 20 (1 SD) HU. Notably, the real adult skull HU was high with a high standard deviation of 245 HU (see Fig. 3b). The STEEV skull HU was comparable to that for the SRS QA phantom. Simple geometry point dose measurements in PLA and StoneFil slabs agreed with RW3 slab phantom within ± 0.2% and ± 0.8% respectively.

### SRS QA phantom design and print

**Fig. 4:**
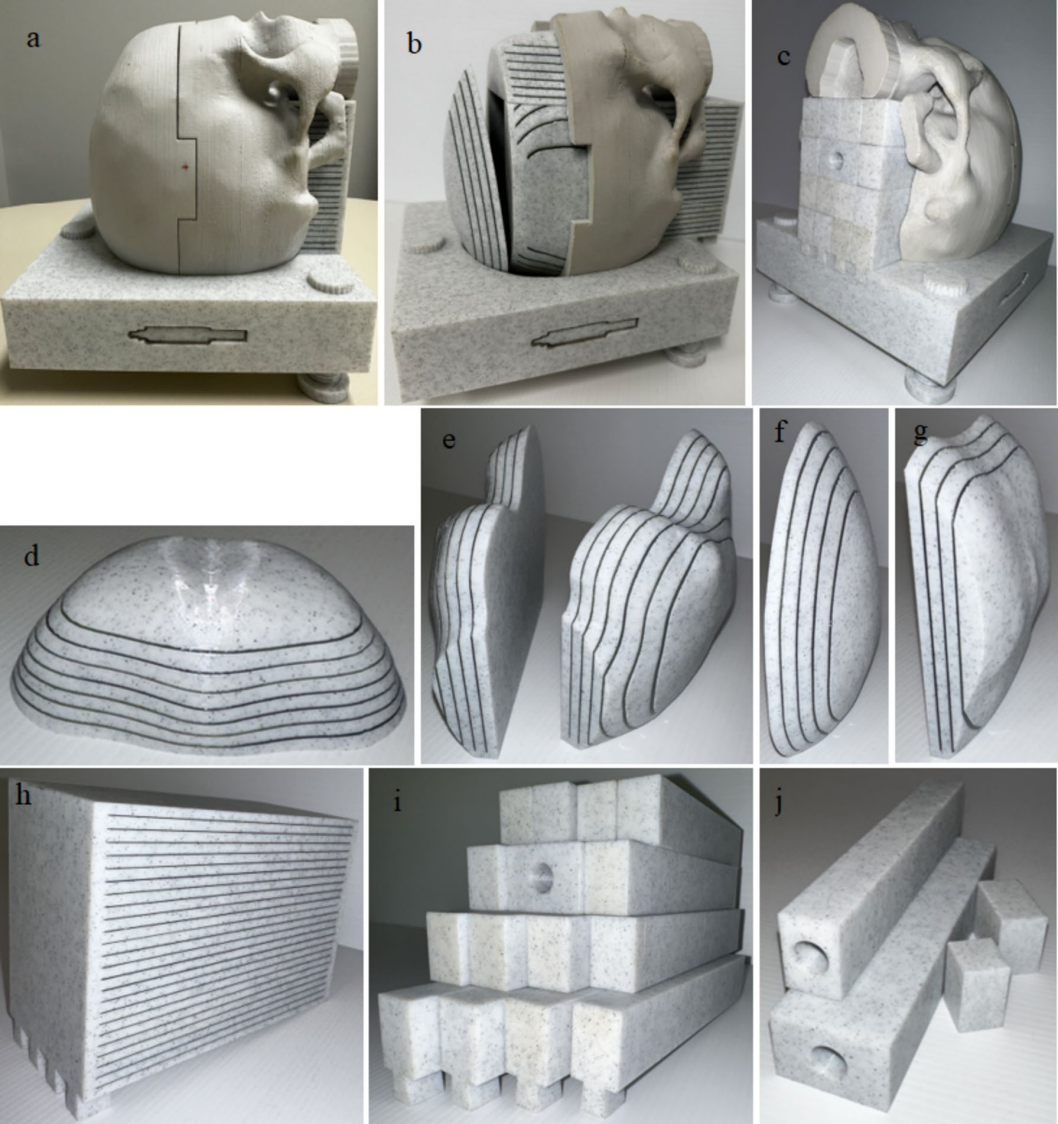
**a** SRS QA phantom **b** SRS QA phantom with the superior skull removed, showing all film inserts **c** SRS QA phantom with ionisation chamber plugs inserted **d** Anterior film insert **e** left and right film inserts **f** superior film insert **g** posterior film insert **h** middle film insert **i** ionisation chamber and microdiamond plugs and **j** plug inserts to increase points of measurements

The phantom has 6 film inserts and several plug inserts as shown in Fig. 4. This configuration enables film measurements at several slots in the transverse, sagittal and coronal planes. The design of the phantom allows for convenient film and point dose measurements in the middle section without requiring the opening of the superior skull part. Peripheral film and middle point dose measurements can be performed simultaneously. Plugs in Fig. 4j allow for more point measurements in the superior-inferior and lateral directions.

### SRS QA phantom quality control

The transverse, sagittal, and coronal film slots were aligned orthogonally within a tolerance of 0.5°, ensuring proper alignment of the film inserts. The inserts were designed to have a snug fit, while still allowing for easy replacement of the of middle film insert and point dose measurement plugs when necessary. Importantly, the capability for image matching and tracking was successfully demonstrated on both the Varian Truebeam and CyberKnife systems.

### End to end testing

The ionisation chamber point dose measurement at the centre of the phantom, delivered on the Varian Truebeam was within 2.5% of that calculated. The film dose results are tabulated in Table 2. Five GTVs were easily assessed in a single CyberKnife plan delivery. However, GTV6 was difficult to assess as it lay at the edge where the middle and superior film inserts met.

**Table 2:** CyberKnife 6 BMT patient specific QA using EBT3 on SRS QA phantom

Target	Insert/plane	Slot	3%/2 mm	5%/1 mm
GTV1	Middle	3	97.7	92.2
		4	98.0	95.6
GTV2	Left	1	99.4	96.9
		2	100.0	100.0
GTV3	Left	2	99.5	92.2
		3	99.3	99.3
GTV4	Left	3	98.0	96.1
		1	96.2	92.0
GTV5	Left	3	91.1	86.7
GTV6	Middle	2	–	–

## Discussion

### Filament assessment

In this study, PLA marble was compared with PLA black and were found to be comparable in terms of HU numbers and by extension was taken to have similar dosimetric properties in megavoltage photon beams. The simple geometry measurements confirmed the dosimetric characteristics of the PLA and StoneFil filaments. PLA marble enabled smooth and consistent printing and has high impact resistance. These qualities make it desirable for the fabrication of chamber plugs, inserts, and film inserts, which are prone to accidental drops during setup.

The middle plugs were printed with 93% PLA infill, which matched the deduced PLA infill calibrations. However, this was not the case with film inserts. Using 93% PLA infill for the film inserts resulted in higher HU (170). Further investigations showed that the perimeter infill could not be adjusted which resulted in a solid perimeter print. Inter-film slot thickness of 4 mm and 1 mm perimeter thickness were small such that 100% infill on perimeter greatly influenced the film insert HUs. Therefore, 83% film infill was determined through trial and error. PLA stoneFil with 100% infill closely matched skull in the STEEV phantom and was within a HU standard deviation of real adult skulls. The large HU standard deviation for real patient skull compared to the printed phantom was expected due to the low-density material sandwiched between the real patient high density skull bone.

### SRS QA phantom design and print

The phantom incorporates several films inserts, that enabling numerous EBT3 film dose measurements. The plug inserts allow several point dose measurements using the CC04 ionisation chamber and microdiamond. This extensive array of measurement points allows for the simulation and evaluation of complex target geometries and configurations during MBT SRS commissioning. The design of the phantom allows for the convenient exchange of the middle film insert and point dose measurement plugs without compromising the integrity of the peripheral film inserts. This feature greatly simplifies the setup process and enhances usability. The snug fit of the bottom part of the middle insert or plugs into the base ensures consistent and reproducible setup and immobilisation. The phantom’s smooth finish and marble look gives the phantom an aesthetic appearance.

### SRS QA phantom quality control

**Fig. 5:**
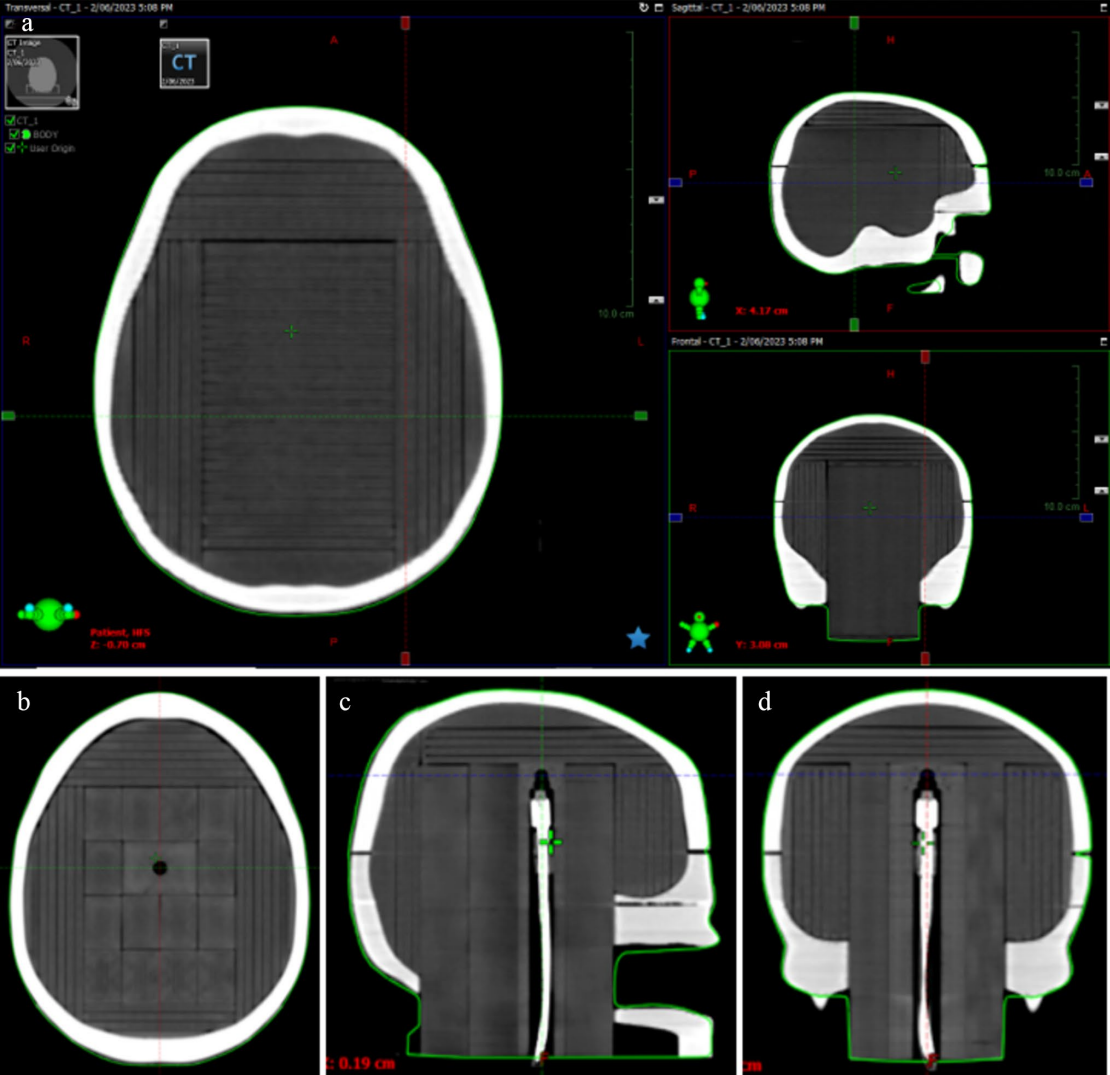
Assessment of precise film locations in transverse, sagittal and coronal planes

The phantom had a snug fit in the base, and the levelling mechanism worked properly. CT scanning of the phantom was performed, and the transverse, coronal, and sagittal orthogonality were assessed, as shown in Fig. 5. This assessment was crucial since films are inserted in film slots corresponding to the planned film planes. Film slots that are not orthogonal could cause erroneous dose assessments as the planned film plane will not align with the film measurement plane. Trackability of the QA phantom was essential for delivering SRS treatments on the CyberKnife, and this was confirmed.

### End to end testing

Point dose assessment using a CC04 ionisation chamber on a 4 MBT planed with the Eclipse Acuros XB dose-to-medium algorithm and delivered on a Varian Truebeam linear accelerator showed that the measured and calculated dose was within 2.5%. The dose deviation was consistent with our department’s independent dosimetry audit, which showed an average deviation of 2.0% for Eclipse Acuros XB dose-to-medium in volumetric modulated arc therapy (VMAT).

Five of the six MBT treatments delivered on the CyberKnife system could be assessed in a single delivery, which took an average of 40 min. This saved at least 2 h that could have been lost from plan re-delivery. The gamma analysis presented in Table 2 demonstrates the effectiveness of evaluating MBT treatments in a single delivery using the SRS QA phantom.

**Fig. 6:**
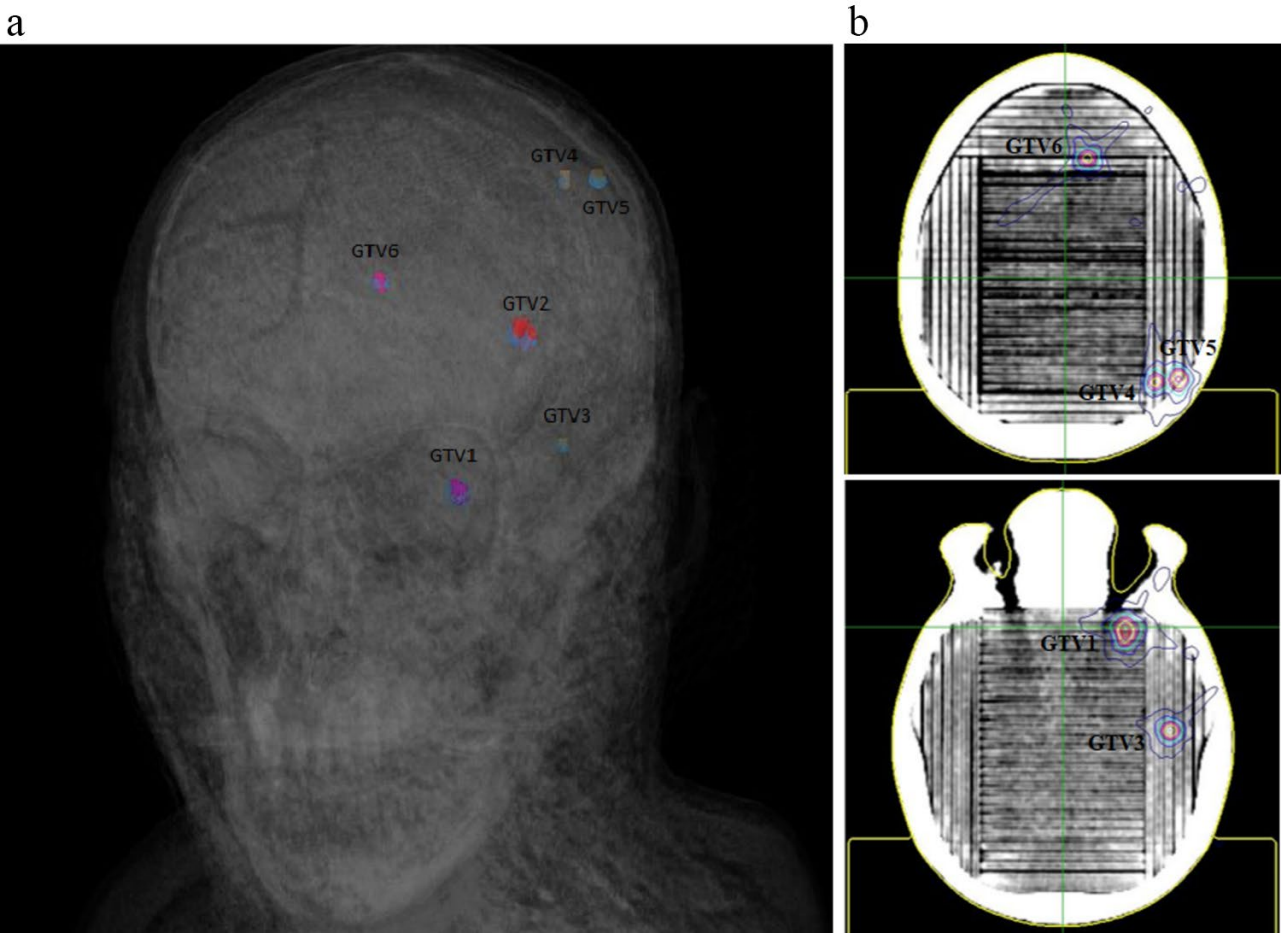
**a** GTV locations on real patient **b** Plan (GTVs) mapped onto the SRS QA phantom

Due to time and resources constraints, our department’s practice is to measure one GTV for patient specific QA, and the result is assumed to represent the other GTVs. Among the GTVs listed in Table 2, GTV5 exhibited a lower pass rate compared to the other GTVs. This low pass rate may have been overlooked if only GTV1-4 were selected for measurement. The inability to measure the 6th GTV (GTV6) could be viewed as a limitation of the phantom. Upon closer examination of GTV6 in Fig. 6, it is evident that the GTV is positioned in a way that makes film dose assessment impossible. The insertion of fiducials allows the phantom to be translated to align the film plane with the centre of the GTV, thereby allowing evaluation of nearly all scenarios in a single plan delivery. However, fiducial insertion was not pursued at this stage as the phantom is earmarked for conventional linear accelerator based MBT SRS commissioning.

Further improvements to the phantom include inserting fiducials, reprinting the inferior skull part to eliminate the observed print defect, and reprint the large point dose measurement insert with reduced percentage infill. These improvements aim to further refine the functionality and accuracy of the phantom.

## Conclusion

3D printing enables the creation of customised dosimetry phantoms. The printed SRS QA phantom offers the advantage of evaluating MBT in a single delivery, providing rapid and comprehensive patient specific QA. This approach not only saves machine time but also conserves associated resources, which can then be allocated towards treating additional patients, developing new techniques, or addressing other pertinent issues as required.
